# Operational failures in general practice: a consensus-building study on the priorities for improvement

**DOI:** 10.3399/BJGP.2023.0321

**Published:** 2024-04-16

**Authors:** Carol Sinnott, Ahmed Alboksmaty, Jordan M Moxey, Katherine I Morley, Sarah Parkinson, Jenni Burt, Mary Dixon-Woods

**Affiliations:** The Healthcare Improvement Studies (THIS) Institute, Cambridge.; The Healthcare Improvement Studies (THIS) Institute, Cambridge.; THIS Labs, Cambridge.; RAND Europe, Cambridge.; RAND Europe, Cambridge.; THIS Labs, Cambridge.; The Healthcare Improvement Studies (THIS) Institute, Cambridge.

**Keywords:** Delphi technique, general practice, operational failures, organisational efficiency, primary health care, quality improvement

## Abstract

**Background:**

System problems, known as operational failures, can greatly affect the work of GPs, with negative consequences for patient and professional experience, efficiency, and effectiveness. Many operational failures are tractable to improvement, but which ones should be prioritised is less clear.

**Aim:**

To build consensus among GPs and patients on the operational failures that should be prioritised to improve NHS general practice.

**Design and setting:**

Two modified Delphi exercises were conducted online among NHS GPs and patients in several regions across England.

**Method:**

Between February and October 2021, two modified Delphi exercises were conducted online: one with NHS GPs, and a subsequent exercise with patients. Over two rounds, GPs rated the importance of a list of operational failures (*n* = 45) that had been compiled using existing evidence. The resulting shortlist was presented to patients for rating over two rounds. Data were analysed using median scores and interquartile ranges. Consensus was defined as 80% of responses falling within one value below and above the median.

**Results:**

Sixty-two GPs responded to the first Delphi exercise, and 53.2% (*n* = 33) were retained through to round two. This exercise yielded consensus on 14 failures as a priority for improvement, which were presented to patients. Thirty-seven patients responded to the first patient Delphi exercise, and 89.2% (*n* = 33) were retained through to round two. Patients identified 13 failures as priorities. The highest scoring failures included inaccuracies in patients’ medical notes, missing test results, and difficulties referring patients to other providers because of problems with referral forms.

**Conclusion:**

This study identified the highest-priority operational failures in general practice according to GPs and patients, and indicates where improvement efforts relating to operational failures in general practice should be focused.

## Introduction

The UK NHS relies on general practice as the bedrock of a functional, cost-effective, and sustainable health service. However, maintaining quality and access has become increasingly challenging as demand outstrips resources and capacities.^1^^,^^2^ It is, therefore, of critical concern that operational failures in primary care — system factors that disrupt workflow and interfere with care provision^3^^,^^4^ — are addressed, as they represent a major drain on capacity,^4^^,^^5^ and negatively affect professional and patient experience.^6^ At least 5% of GPs’ time during clinical sessions alone (which is intended for direct clinical care) appears to be spent on resolving operational failures,^7^ but this figure does not include the time GPs or their colleagues spend on dealing with system defects when patients are not in clinic.

Multiple different types of operational failures affect the work of GPs.^8^ They include, but are not limited to:

- problems in care coordination, both within practices and across sectors and institutions;
- interruptions and disruptions;
- missing materials and equipment (for example, urinanalysis containers, thermometer probe covers, etc.);
- problems with information sharing, including test results, findings of investigations, and discharge letters from, and with, other parts of the healthcare system;
- challenges in making referrals; and
- suboptimal IT, computing resources, and electronic health records (EHRs).^5^^,^^8^

Many of these failures may be tractable^9^ but, given their number and range, it is critical to determine which ones stakeholders see as being most important before embarking on improvement efforts. To address this need, the authors sought to establish consensus across GPs and patients on the most important operational failures to prioritise for future improvement work in general practice.

**Table:** How this fits in

Operational failures in primary care are system factors that interfere with GPs’ work and cause problems in care. Using data from three recent studies on operational failures in general practice, this study identified the operational failures seen as most important to target for improvement actions by GPs and patients. The highest scoring failures related to inaccuracies in patients’ medical notes, unavailable or missing test results, and difficulties referring patients to other healthcare services because of problems with referral forms. These findings indicate where improvement efforts relating to operational failures in general practice should be focused.

## Method

### Study design

Two consecutive modified Delphi exercises that sought consensus on the most important operational failures in general practice to target for improvement were conducted. The first involved GPs and the second involved patients. The surveys were conducted between February and October 2021 using Thiscovery (https://www.thiscovery.org/), a secure online research and development platform developed by The Healthcare Improvement Studies (THIS) Institute.

**Figure 1.**
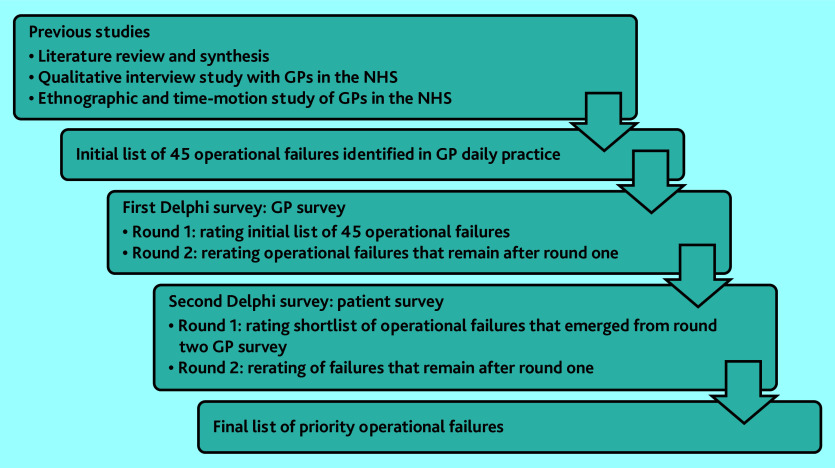
Overview of the data collection steps throughout the study.

Operational failures were defined, based on previous work, as problems in the supply of equipment, materials, or information needed to complete a work task, or as an interruption that takes the individual’s attention away from a work task they are already doing.^4^^,^^7^ For the first Delphi exercise, an initial list of 45 operational failures (Supplementary Table S1) was informed by three previous studies^5^^,^^7^^,^^8^ on the topic in general practice. GPs participated in two survey rounds to reach consensus on the operational failures they considered a priority. The findings of this exercise formed a shortlist of operational failures that was the basis of a Delphi exercise in which patients participated, which also comprised two rounds. Figure 1 illustrates an overview of the data-collection steps throughout the study.

### Survey structure

Each survey presented a list of operational failures to be rated in terms of priority for improvement on a nine-point scale, ranging from one (not at all important) to nine (extremely important). Each operational failure was accompanied by a drop-down description and example to ensure participants had a consistent understanding of what was meant. Supplementary Boxes S2 and S3 show examples of the surveys. Participants were also invited to provide reflections on the operational failures as free text.

In the second round of each Delphi exercise, participants were provided with their own rating, the median rating of the entire group for each operational failure, and the spread of responses from the group in the form of a graph. They were invited to rerate each failure and explain their decision if they changed their rating. Each survey included questions about participants’ demographics and, in the case of GPs, professional roles.

### Patient and public involvement and engagement

The Cambridge University Hospitals Patient and Public Involvement and Engagement panel reviewed the patient-facing recruitment materials and surveys. The panel’s suggestions for improving clarity, survey layout, and wording were addressed. The panel’s recommendation that participants be offered the opportunity to complete the survey with the assistance of a trusted friend or carer was also implemented.

### Eligibility and recruitment

Eligibility criteria included the ability to provide informed consent, understand and speak English, and have internet access (with support if needed). GP participants needed to be actively working in an NHS general practice, and patient participants had to be registered with an NHS general practice.

GPs were recruited using convenience and snowball techniques, as outlined by Green and Thorogood.^10^ Invitations were sent to GPs via the email lists of their professional bodies, the networks of THIS Institute and its collaborators, and via social-media platforms, such as Facebook groups, that only have GP membership. A certificate that could be used as evidence of continuing professional development was offered to GPs.

To recruit patients, the authors partnered with Healthwatch England, an independent national champion for people who use health and social care services. A local Healthwatch team in each of the seven NHS regions sought to recruit approximately six people with diversity in terms of age, gender, and ethnicity. This sample size was decided after reviewing relevant evidence on the numbers needed for Delphi-type exercises.^11^^,^^12^

To minimise attrition between rounds, a £25 online shopping voucher was offered to each participant who completed both rounds (both patients and GPs) as a token of appreciation.

### Data collection

The surveys were hosted on the Thiscovery platform, which was accessible through a direct link embedded in invitation emails. The Thiscovery landing page provided a summary of the study and a participant information sheet. To take part, potential participants registered with Thiscovery, answered three eligibility questions, and then electronically signed a consent form before commencing the survey. The questions were whether they had the ability to understand and speak English; had access to a device with internet access (for example, PCs, phones, tablets); and specific eligibility criteria depending on whether they were a GP or a patient. GPs were asked to confirm that they were currently working in general practice in the NHS and patients were asked to confirm that they were currently registered with a GP surgery.

### Data analysis

Survey responses were included in the analysis if a participant responded to at least one survey question. The free-text comments from each survey were collated and analysed qualitatively using a thematic approach.^10^ Ethnicity data were compiled using the preferred style outlined by the UK Government.^13^

After each round, rating scores were used to calculate a measure of central tendency (median), a measure of distribution (interquartile range), and the proportion of responses for each score for each operational failure. The threshold for consensus that an operational failure was important was defined a priori as:

- failures achieving a median score for importance of ≥7; and
- group consensus in which 80% of responses fell within three ratings, one of which was the median.

Following feedback from GP participants in round one that the survey was too long, a pragmatic research decision was made to reduce the number of items presented in round two by also removing all failures with a median score of <7 for importance, regardless of the level of consensus.

The operational failures that met both the importance and consensus criteria in the second GP survey were presented to patients for rating. Those operational failures that achieved both the importance and consensus criteria after the second patient survey were deemed the outcomes of the study.

## Results

### GP Delphi exercise

Sixty-two GPs participated in the round-one GP survey. More than two-thirds were female (68%) or aged >40 years (61%). Of those who answered a question on ethnicity, 28% (*n* = 11) were of minority ethnicity, as classified by the UK Government.^13^ More than half (55%) had at least 11 years of experience as GPs in the NHS, and more than a quarter (26%) reported having more than 20 years’ experience. Just fewer than half (48%) were partners in their practices. The retention rate of GPs through to round two of the survey was 53% (*n* = 33), with no marked differences in characteristics between those GPs who were retained and those who were lost.

Round one of the GP Delphi exercise produced consensus on the high importance of six of the 45 operational failures listed; these six failures were not presented to GPs again in round two (Table 1). Fourteen failures with a median score of <7 for importance were also excluded after round one, leaving 25 failures to be rerated by GPs in round two. After round two, another eight failures met the shortlisting criteria, leading to 14 failures being prioritised by GPs overall (Table 1). The full list of failures, their importance, and consensus ratings are provided in Supplementary Table S1. The three failures with the highest scores related to:

- the EHR not functioning properly (median 9; consensus 89.6%);
- requests from hospitals for GPs to organise investigations or referrals for patients that could have been organised by the hospital team themselves (median 9; consensus 89.5%); and
- patients attending GPs, when they had not received an appointment with an external service, to find out when the appointment was to take place or to ask the GP to refer them back to the external service if the appointment had been missed (median 9; consensus 84.2%).

**Table 1.** Median score for importance and level of consensus for the 14 operational failures meeting shortlisting criteria in the GP Delphi survey

**Question/statement**	**Round one**		**Round two**		**Change in median**	**Change in consensus, %**
	**Median**	**Consensus, %**	**Median**	**Consensus, %**		
The EHR stops functioning properly, functions very slowly, or crashes while you are using it to complete a task^a^	9	89.6		—	—	—
A patient has not been told the results of their hospital investigations, so consults with you to get the results instead^a^	8	80.0		—	—	—
A letter about a patient that you are expecting, or need to receive, from a hospital or other service has not been received^a^	8	88.9		—	—	—
Not enough time is allocated in your schedule for your clinical paperwork^a^	8	83.7		—	—	—
You are unable to contact other healthcare professionals by phone when you need them^a^	8	86.4		—	—	—
You cannot find the information you need on a patient in the EHR because the design of the record is complicated or not intuitive^a^	8	83.3		—	—	—
A letter about a patient that is sent to you by a hospital or other service requests you to arrange investigations or referrals that could have been arranged by the sender themselves	9	75.6	9	89.5	0	13.9
A patient has not been informed of the date of their appointment with another service, so attends you to try to find out when it is or to request referral back to that service if the appointment was missed	9	68.9	9	84.2	0	15.3
You experience problems making telephone contact because the patient’s contact details in the EHR are incorrect	8	77.1	8	85.7	0	8.6
You are unable to review a test result because of problems in the laboratory or practice IT system	8	68.2	8	84.2	0	16.0
You are unable to review a test result because it is missing	8	79.5	8	81.0	0	1.5
You have problems referring your patient to hospital or other healthcare professionals, because you do not have the most up-to-date referral template or the template requires a lot of extra information that you don’t have to hand	7	75.0	7	89.5	0	14.5
The coding of patient information in the EHR is inaccurate or missing. (Coding is a process of allocating standardised terms from a recognised classification system to a patient’s medical notes)	7	75.0	7	81.8	0	6.8
You find it difficult to sign off test results because you have been allocated results for tests that you did not order, because you are not familiar with the patient who had the tests, or because the reason for the test is unclear	7	64.4	7	81.0	0	16.6

a *Consensus achieved in round one, so excluded from round two for rerating. EHR = electronic health record.*

### Patient Delphi exercise

**Figure 2.**
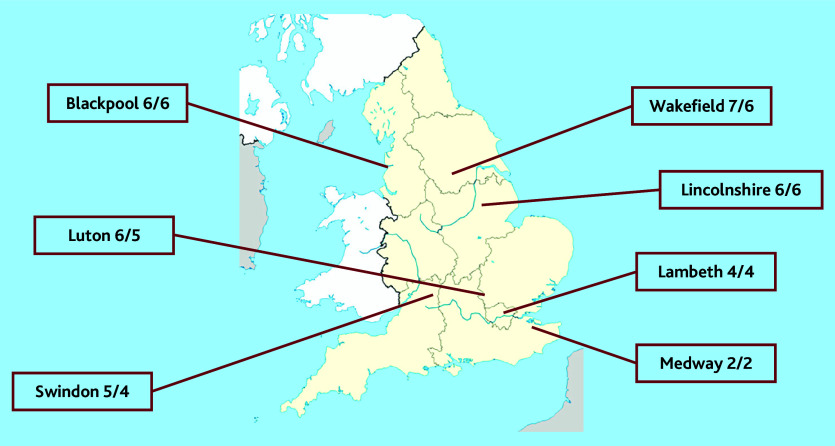
Number of patient participants recruited and retained (*n*/*n*) by each local Healthwatch group. ^a^One responder did not indicate which Healthwatch group they were part of.

Patient participants were recruited from seven local Healthwatch groups (Figure 2). A total of 37 participants completed round one: the majority were female (64%), aged >40 years (55%), and attended practices in urban areas (75%). Of those who answered a question on ethnicity, 29% (*n* = 10) were of minority ethnicity. The retention rate through to the second round was 89% (33/37).

The 14 operational failures prioritised by GPs were presented to patient participants for rating; as they were all rated as highly important (median score ≥8) in round one (Table 2), they were all presented for rerating in round two. After round two, 13 operational failures achieved the importance and consensus criteria, and were included in the final list (Box 1). The three failures with the highest patient scores were inaccuracies or missing information in a patient’s medical notes on the EHR (median 9; consensus 96.9%), a GP being unable to review a patient’s test result because the result was missing (median 9; consensus 93.8%), and problems referring a patient to hospital or other healthcare service because the right form was not available, was badly designed, or required information that the GP did not have to hand (median 9; consensus 84.8%) (Table 2).

**Table 2.** Median scores and level of consensus for the 14 operational failures included in the patient Delphi exercise

**Question/statement**	**Round one**		**Round two**		**Change in median**	**Change in consensus, %**
	**Median**	**Consensus, %**	**Median**	**Consensus, %**		
The way your medical notes are classified or coded on the EHR is inaccurate or information (for example, a diagnosis) is missing^a^	9	91.9	9	96.9	0	5
Your GP is unable to review your test result because it is missing	9	75.7	9	93.8	0	18.1
Your GP has problems referring you to hospital or other healthcare services because the right form is not available, is badly designed, or requires a lot of extra information they do not have to hand^a^	8.5	94.4	9	84.8	0.5	−9.6
You have not been told the date of your appointment with another healthcare service, so you see your GP to try to find out when it is, or to request referral back to a service because the appointment was missed	9	73.0	9	84.4	0	11.4
Your GP cannot find the information they need for you on their computer because the design of the EHR is too complicated	9	75.7	9	81.8	0	6.1
Your GP is unable to review your test result because of problems in the laboratory or practice computer system^a^	8	91.9	9	81.2	1	−10.7
The EHR on the computer stops functioning properly, functions very slowly, or crashes while your GP is using it to complete a task^a^	8	91.9	8	93.9	0	2
A letter about you that your GP is expecting to receive from a hospital or other healthcare service has not been received^a^	8	86.1	8	93.5	0	7.4
Your GP experiences problems making telephone contact with you because your contact details on the computer are incorrect	8	75.7	8	87.5	0	11.8
Your GP is unable to contact other healthcare professionals by phone when they need them (for example, district nurses, consultants, emergency response teams, etc.)^a^	8.5	88.9	8	84.8	−0.5	−4.1
Not enough time is allocated in your GP’s schedule for their clinical paperwork (for example, repeat prescribing, reading letters, writing letters, filling in forms, etc.)^a^	8	86.1	8	81.8	0	−4.3
A letter about you that is sent to your GP by a hospital or other healthcare service asks your GP to arrange tests that could have been arranged by the sender themselves	8	64.9	8	81.2	0	16.3
Your test results are sent to a GP who is not your usual doctor, making it difficult for them to sign off your results	8	78.4	8	81.2	0	2.8
You have not been told the results of your hospital tests, so you see your GP to get the results instead	8	75.0	8	78.1	0	3.1

a *Consensus achieved in round one. EHR = electronic health record.*

**Box 1.** Free-text responses relating to the most highly scored operational failures

**Operational failure**	**Example GP entry**	**Example patient entry**
Inaccuracies or missing information in a patient’s medical notes on the electronic health record	*‘High ratings from others prompted me to reflect* *deeper on my initial high rating. Codes, especially* *diagnoses, increasingly drive care — algorithms* *imposed on us, QOF pop-ups, etc. Problems with* *this system really do generate a lot of unnecessary* *consults and tasks, take time to explore and fix, or* *confuse consultations where a wrong code becomes* *the focus. Colleagues who don’t know how to sort* *codes and allow records to deteriorate can place* *a major burden on those who do — it is a constant* *process of tending!’* (GP2.R2)	*‘It is vital, especially in patients with* [a] *complex* *medical history, that information is accurate and up* *to date, as this could lead to misdiagnosis or wrong* *treatment/procedures being followed. Patients are* *more than their diagnosis, as all are interlinked with* *the wellbeing of the patient.’* (P2.R4)
A GP is unable to review a patient’s test result because the result is missing	*‘We need a more reliable system for telling us results are* *“missing” before we prioritise finding them.’* (GP2.R3)=	*‘This is out of the GP’s control but has a huge impact* *on how they can support and offer the right care for* *the patient. It also has an impact on* [the] *patient’s* *time and appointment availability, leading to* *“wasting” both GP and patient time if results are not* *available for the GP at a consultation.’* (P2.R16)
Problems referring a patient to hospital or other healthcare service because the right form is not available, is badly designed, or requires a lot of extra information the GP does not have to hand.	*‘I could get administrative colleagues to help with* *this task, but it still creates a lot of work.’* (GP1.R2)	*‘There does need to be much more streamlining in* *relation to referrals, sharing medical records, and* *using the same systems would assist this — and* *perhaps more self-referrals put in place to ease strain* *on the GP.’* (P1.R2)

*QOF = Quality and Outcomes Framework.*

### Free-text results

GP participants entered 132 free-text comments: 99 were in relation to specific operational failures, 22 were overarching comments on operational failures, and 11 were on the survey format. Patients entered 166 free-text comments: 113 on specific operational failures, 26 overarching comments on operational failures, and 27 on the survey format.

GPs’ comments were organised into three overarching themes:

- the extent to which addressing operational failures was within GPs’ control: *‘Problems that are related to the practice set-up are easier to address,* [for example] *making sure consultation rooms are properly equipped, whereas those dependent on outside organisations are more difficult,* [for example] *requests coming from secondary care. The latter may be easy to fix in principle, but not in practice.’* (GP.1R4);
- how technology can make GPs’ work more efficient in many regards, but only when it works: *‘All the problems are magnified simply by high workload and competing demands on my time, and what has become unrealistic expectations from GPs. IT systems have made my job immeasurably easier in many respects, but only when they work!’* (GP.1R9); and
- the bidirectional negative relationship between high workload and operational failures in general practice: *‘I left daytime GP for many reasons but the constant inability to do my job by factors outwith my control absolutely wore away at me and contributed to my decision to leave. I couldn’t take it any more.’* (GP1.R7)

Patients’ responses were also organised into three overarching themes:

- the importance of seamless communication at the primary–secondary care interface: *‘It is evident that some third-party providers, including hospitals, do not check the records they send to GPs. It becomes a sort of “telephone/message whispers game”, where records become miscommunicated as the information is passed between practice and provider.’* (P1.R14);
- the requirement for well-designed IT to support the care they receive: *‘I notice most electronic systems seem to be over-complicated using databases/software that is not user friendly for staff or patients.’* (P1.R14); and
- the benefits of continuity of care in mitigating against the risk of operational failures and ensuring consultations are used effectively: *‘The most important aspect is being able to always see the particular GP you wish to. Otherwise, far too much time is taken up with telling your back story over and over again, with the risk that the emphasis the GP puts on different aspects of your case* [is] *less salient.’* (P1.R19)

Free-text responses relating to the most highly scored operational failures are shown in Box 1. Patients also called for further research on failures relating to access to general practice including delays when trying to make appointments, the role of technology as an additional barrier to access, and problems getting to see one’s preferred GP.

## Discussion

### Summary

After engaging GPs and patients in prioritising operational failures in NHS general practice, this study identified 13 priorities for improvement. The three highest scoring failures related to inaccuracies or missing information in medical records, GPs being unable to review a patient’s test result because it was missing, and problems referring a patient to hospital or other healthcare service.

### Strengths and limitations

The generalisability of the findings is supported by diversity among participants with a wide range of ethnicities, ages, and levels of professional experience from regions across England. Although the content of the patient surveys had the potential to be complicated, the retention of patient participants was high, potentially as a result of the patient and public involvement and engagement work, along with user testing, which was done to ensure that patient-facing material was clear and well presented.

Despite the option of support, the online nature of the exercise may have risked excluding some possible participants, including those experiencing digital poverty or low digital literacy.^14^ In addition, as this study was undertaken during the COVID-19 pandemic, retaining GP participants may have been adversely affected by the competing demands of staff absences and COVID-19 vaccination pressures^15^ — in this context, however, a retention rate of 53% was perhaps satisfactory. Although there is no guidance on what constitutes an acceptable retention rate in Delphi studies, around 80% for each stakeholder group is a typical target.^16^ The authors felt that a sample size of 15–30 GPs would be appropriate for this study, but decided to over-recruit GPs in anticipation of higher levels of attrition, given the context of the pandemic. Steps were also taken to encourage participation in the second round — for instance, by shortening the survey and offering vouchers for participation. The similarity between the demographic characteristics of GPs who were retained and those who did not complete round two was reassuring, but it is not possible to determine whether the results would have differed if a higher proportion of GPs had been retained.

After round one, GPs fed back that the survey was too long. The authors addressed this by excluding failures that did not meet the threshold for importance after round one, even if there was not consensus around the low scores. It is possible that some GPs might have increased their importance ratings for these excluded failures had they seen them again in round two. However, none of the failures presented to GPs in round two achieved higher importance scores; only consensus ratings improved in round two, making it less likely that any of the excluded failures would have met the prioritisation criteria, even if they had been presented to GPs again in the second-round survey.

Patients were only shown the operational failures that had already been identified by GPs as being important. This approach was taken to reduce participant burden by presenting patients with only those failures that were having the biggest impact on GPs’ work. However, had patients been presented with the full list of operational failures their prioritisation may have diverged further from that of the GPs.

The free-text data was a small, but useful, component of the study, offering insight into the reasons for different scores and patients’ opinions on the need for research on operational problems relating to general practice access.

### Comparison with existing literature

This study builds the evidence base on operational failures in NHS general practice by drawing together the findings of an earlier systematic review by some of the authors,^5^ an interview study with NHS GPs,^8^ and a mixed-method ethnographic study of NHS GPs.^7^ The top priority for GPs was having access to an efficient, dependable EHR, but failures relating to inaccurate or missing information in EHRs also scored highly. This finding is consistent with other evidence showing that GPs rely on EHRs, not only as a repository for patient-related information, but also to support clinical decision making, and for the coordination of services, information exchange, administration, audit, and recall of patients.^17^^,^^18^ Problems relating to EHRs extend beyond frustrating health professionals: they can also lead to serious errors in decision making and prescribing, delays in care, and other patient harms.^19^ Patients’ priorities differed from those of GPs; understandably, patients are less affected by day-to-day failings — for example, in relation IT and the EHR — and the compensatory labour undertaken by GPs may not have been visible to patients. However, this study’s findings confirm that patients are sensitive to operational failures in general practice, including — but not limited to — problems relating to information handling and technology that potentially affect their own experiences of continuity, fragmentation, and coordination of care.

### Implications for research and practice

Operational failures disrupt health professionals, introduce unnecessary pressures, cause delays, and introduce risks for patient safety, as well as the patient and staff experience. This study has identified the highest-priority operational failures in general practice, and the findings offer clear signals as to where future improvement efforts in general practice might be focused; the final list of 13 operational failures can be used by researchers, improvers, commissioners, and practices to inform pragmatic, high-impact quality-improvement activities.

Notably, this study took place during the COVID-19 pandemic, when practices experienced sudden increases in their dependence on digital communications, remote consultations, and expectations for primary care to carry out functions that had previously been undertaken in secondary care; the enduring relevance of these shifts supports the importance of this study in planning priorities for improvement. There are now fewer full-time-equivalent GPs working in the NHS than before the COVID-19 pandemic, while patient need is increasing.^2^^,^^20^ Trying to do more with less is leading to record-high levels of work-related stress among GPs;^20^ at the same time, the proportion of patients who rate their experience of general practice as ‘good’ is deteriorating rapidly.^21^ Reversing negative trends will require, among other things, improvements in operational and administrative processes that currently lead to waste and stress. This study is important in identifying GPs’ and patients’ priorities for improvement.

The large-scale system improvement that is needed to address the challenges effectively is unlikely to be possible at individual-practice level, and attempting to do so may, paradoxically, add further stress to an already overburdened system. As an example, dealing with problems in information handling and technology systems that straddle institutional boundaries is an impossible ask of individual practices. An alternative approach would be to address operational failures collaboratively and at scale through a learning-system approach that includes patients, diverse staff groups, primary care networks, and the power of the NHS’s routinely collected general practice data to codesign, implement, and test changes that aim to improve operational processes and practice efficiency.^22^ The Care Quality Commission could, potentially, be engaged in such processes to ensure that learning and innovation are captured through regulatory processes, as appropriate. System-level changes have the potential to produce new operational challenges for GPs — for example, by further fragmenting care or duplicating effort — so high-quality design, implementation, and evaluation will be critical, as has happened in the area of skill-mix, for example.^23^

Future analyses should also consider whether patients attending, and GPs serving in, practices in areas of deprivation have differing priorities for improvement.

## Data

This dataset is not publicly available.
